# Describing small-angle scattering profiles by a limited set of intensities

**DOI:** 10.1107/S1600576722006598

**Published:** 2022-08-30

**Authors:** Thomas D. Grant

**Affiliations:** a Department of Structural Biology, Jacobs School of Medicine and Biomedical Sciences, University at Buffalo, NY 14203, USA; European Molecular Biology Laboratory, Hamburg, Germany

**Keywords:** small-angle scattering, indirect Fourier transform, solution scattering, pair distribution function

## Abstract

Small-angle scattering (SAS) probes the size and shape of particles at low resolution through the analysis of the scattering of X-rays or neutrons passing through a solution of particles. One approach to extracting structural information from SAS data is the indirect Fourier transform (IFT). The IFT approach parameterizes the real-space pair distribution function [*P*(*r*)] of a particle using a set of basis functions, which simultaneously determines the scattering profile [*I*(*q*)] using corresponding reciprocal-space basis functions. This article presents an extension of an IFT algorithm proposed by Moore [ *J. Appl. Cryst.* (1980), **13**, 168–175] which used a trigonometric series to describe the basis functions, where the real-space and reciprocal-space basis functions are Fourier mates. An equation is presented relating the Moore coefficients to the intensities of the SAS profile at specific positions, as well as a series of new equations that describe the size and shape parameters of a particle from this distinct set of intensity values. An analytical real-space regularizer is derived to smooth the *P*(*r*) curve and ameliorate systematic deviations caused by series termination. Regularization is commonly used in IFT methods though not described in Moore’s original approach, which is particularly susceptible to such effects. The algorithm is provided as a script, denss.f it_data.py, as part of the *DENSS* software package for SAS, which includes both command line and interactive graphical interfaces. Results of the program using experimental data show that it is as accurate as, and often more accurate than, existing tools.

## Abstract 2

An indirect Fourier transform method is presented which describes a solution scattering profile from a reduced set of intensities. Equations are derived to fit the experimental profile using least squares and to calculate commonly used size and shape parameters directly from the reduced set of intensities, along with associated uncertainties. An analytical equation is derived enabling regularization of the real-space pair distribution function. Convenient software is provided to perform all described calculations.

## 1. Introduction and overview

Small-angle scattering (SAS) yields structural information at low resolution about the size and shape of particles in solution. X-rays or neutrons scattering from freely tumbling particles in solution exhibit rotational averaging in reciprocal space, resulting in isotropic scattering profiles collected on 2D detectors. This rotational averaging results in the loss of information describing the 3D structure of the particle. The scattering of a molecule *I*(*q*), where *q* is the momentum transfer [*q* = (4π/λ)sinθ, where θ is half the scattering angle and λ is the wavelength of the incident radiation], is determined by its 3D scattering length density function, and thus SAS profiles can be calculated directly from known atomic structures. However, due to the spherical averaging of the intensities, the inverse problem of calculating a unique 3D structure from SAS profiles is not possible. Nonetheless, structural information describing global properties of size and shape can be obtained through analysis of the SAS profile.

While unique 3D real-space information cannot be obtained directly from a SAS profile, a Fourier transform of the reciprocal-space intensity profile yields the set of pair distances in the particle, known as the pair distribution function or *P*(*r*). However, due to limitations caused by the termination of higher-order scattering data to a finite *q* range, uncertainties in intensity measurements and systematic errors, direct calculation of the Fourier transform yields *P*(*r*) functions with large systematic deviations (Glatter, 1977 ▸; Moore, 1980 ▸; Hansen & Pedersen, 1991 ▸; Svergun, 1992 ▸; Svergun & Pedersen, 1994 ▸). One popular approach to extracting this structural information from SAS profiles is the indirect Fourier transform (IFT) proposed by Glatter (1977 ▸). In this approach, a set of basis functions is used to parameterize the *P*(*r*) function. The weights of these basis functions are then adjusted to optimize the fit of the corresponding intensity function to the experimental scattering profile.

One such IFT algorithm proposed by Moore (1980 ▸) takes advantage of information theory (Shannon, 1948 ▸) to describe a set of basis functions defined by the maximum particle dimension *D*. Moore uses a trigonometric series to define a function *Q*(*r*) = *P*(*r*)/*r*. This definition resulted in a convenient relationship between the real-space *Q*(*r*) and the reciprocal-space *U*(*q*) = *qI*(*q*), where the two are Fourier mates. Key to Moore’s approach (and other IFT methods; Glatter, 1977 ▸; Svergun, 1992 ▸) is that the coefficients of the series terms define both the real-space and reciprocal-space profiles, using the appropriate basis functions. Least squares can be used to determine the coefficients and the associated standard errors by minimizing the fit to the experimental scattering profile (full details are given in Section S1 of the supporting information). This approach has the advantage of providing the necessary information on the variances and covariances of the coefficients to determine the errors on each coefficient. Moore showed, using Shannon information theory, that the number of coefficients that can be determined from the data is the number of independent pieces of information that the data are able to describe about the particle. Moore derived a series of equations relating the coefficients to commonly used SAS parameters such as the forward scattering intensity *I*(0), the radius of gyration *R*_g_ and the average vector length 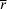 , along with error estimation for each parameter. One advantage of Moore’s approach over others is that a separate regularizing function is not explicitly required to smooth the *P*(*r*) curve due to the use of the sine series (Moore, 1980 ▸). However, in practice with experimental data, it has been found that Moore’s approach is often more susceptible to large oscillations in the *P*(*r*) curve due to series termination (Svergun & Pedersen, 1994 ▸; Hansen & Pedersen, 1991 ▸), probably because of the lack of a regularizing function. Such regularizing functions have been shown to be effective at smoothing the *P*(*r*) curves calculated using Moore’s approach (Tully *et al.*, 2021 ▸; Rambo, 2021 ▸).

Here we extend Moore’s derivation to relate the Moore coefficients to specific intensity values such that each term in the series is now weighted by a corresponding intensity, termed *I*_*n*_ (Section S1 in the supporting information). We present equations for calculating a variety of commonly used SAS parameters and their associated errors from the *I*_*n*_ values. Additionally, we derive a modified equation for least-squares minimization taking into account an analytical regularization of the *P*(*r*) curve. We provide open-source software with convenient interfaces for performing all of the presented calculations, including a novel approach to estimating parameters sensitive to systematic errors. Finally, we describe the results using both simulated and real experimental data and compare with current state-of-the-art software tools.

## 2. Theoretical background

### 2.1. Extension of Moore’s IFT

Moore’s use of Shannon information theory to define *I*(*q*) resulted in a selection of *q* values, namely *q*_*n*_ = *n*π/*D*, termed ‘Shannon channels’ (Feigin & Svergun, 1987 ▸; Svergun & Koch, 2003 ▸; Rambo & Tainer, 2013 ▸). The intensities at *q*_*n*_, *i.e.* *I*_*n*_ = *I*(*q*_*n*_), therefore become important values as they determine the Moore coefficients *a*_*n*_ and thus similarly can be used to describe completely the low-resolution size and shape of a particle obtainable by SAS. In Section S1 we derive the mathematical relationship between *I*_*n*_ and *a*_*n*_ which results in the following general equation for *I*(*q*) as a function of the intensity values at the Shannon points: 

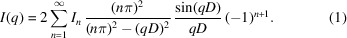

Defining basis functions *B*_*n*_ as 

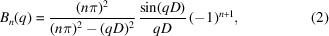


*I*(*q*) can now be expressed as a sum of the basis functions *B*_*n*_ weighted by the intensity values at *q*_*n*_, 

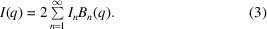




**Figure 1:**
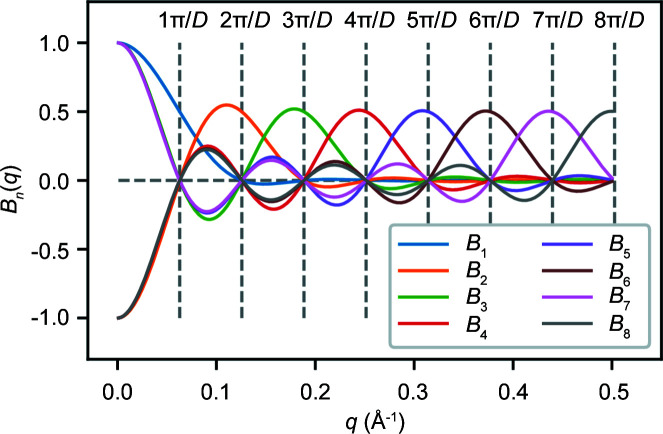
A plot of reciprocal-space basis functions *B*_*n*_ for any particle of size *D* = 50 Å. Vertical dashed lines show the locations of the Shannon points *q*_*n*_.

As in Moore’s original approach, the *B*_*n*_ functions are determined by the maximum dimension of the particle *D*. *B*_*n*_ values for *D* = 50 Å are illustrated in Fig. 1 ▸. The *P*(*r*) function can be represented using the series of *I*_*n*_ values as 

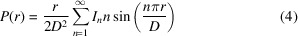

(Section S1) or by defining real-space basis functions *S*_*n*_ as follows: 

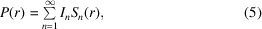




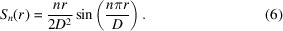

Least squares can be used to determine optimal values for each *I*_*n*_ from the oversampled experimental SAS profile, along with error estimates for each, taking into account the variances and covariances of the coefficients. These terms can then be used to calculate the corresponding *I*(*q*) and *P*(*r*) curves using equations (1) and (4) and the associated errors (Section S1).

The maximum particle dimension *D* is required for determining the *q*_*n*_ values associated with the *I*_*n*_ values. Estimates for the true value of *D* that are too small will result in *B*_*n*_ values that lack sufficiently high frequencies for the adequate reconstruction of *I*(*q*). Estimates of *D* that are too large will result in overfitting the data. Moore found that testing increasing values of *D* yielded improved fits to the experimental *I*(*q*) function and used χ^2^ (Section S1) to estimate the true value of *D* by selecting the smallest *D* value that minimizes χ^2^ while avoiding larger *D* values that result in overfitting (Moore, 1980 ▸). An alternative method is to estimate *D* from the *P*(*r*) curve by first guessing a reasonable value for *D*, such as 3.5*R*_g_ or larger, fit *I*(*q*) and calculate the *P*(*r*) curve, and then estimate the true value of *D* on the basis of where *P*(*r*) gradually falls to zero.

### 2.2. Derivation of parameters from *I*_*n*_ values

Similarly to what Moore described for the *a*_*n*_ coefficients, since the *I*_*n*_ values contain all the information present in *I*(*q*), quantities that can be derived from *I*(*q*) can also be derived directly from the *I*_*n*_ values. For example, to determine the forward scattering intensity *I*(0), we take the limit of equation (1) as *q* approaches zero to yield 

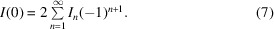

**Figure 2:**
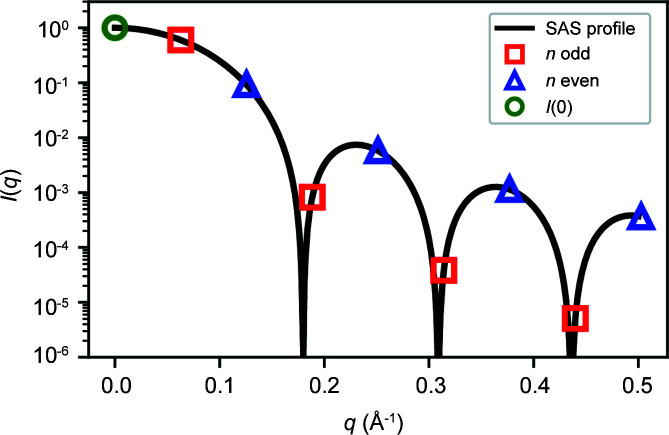
A plot of an example scattering profile, showing the relationship of *I*_*n*_ values and *I*(0). Odd *I*_*n*_ values are shown as red squares, while even *I*_*n*_ values [which have a multiplication factor of −1 in equation (7)] are shown as blue triangles. Equation (7) states that twice the total sum of the red squares and (negative) blue triangles is equal to the forward scattering *I*(0), shown as a green circle.

Equation (7) demonstrates a simple relationship between the forward scattering of a particle and the *I*_*n*_ values. Note that the particle dimension *D* is not explicitly present in equation (7). Fig. 2 ▸ illustrates the relationship between the *I*_*n*_ values and *I*(0).

The forward scattering of a particle is not directly measured in an experiment due to its coincidence with the incident beam and is thus typically estimated as an extrapolated value from low-*q* data points or by integration of the *P*(*r*) function. Equation (7) provides an alternative method of measuring the forward scattering of a particle directly from the data through the sum of the *I*_*n*_ values. While equation (7) is defined as a sum from *n* = 1 to infinity, typical experimental setups only provide data for the first 10–30 Shannon channels, depending on the size of the particle. Thus in practice equation (7) yields an estimate of the forward scattering rather than an exact measurement. However, since the vast majority of the scattering intensity present in the profile occurs within these 10–30 Shannon channels, equation (7) should provide an accurate estimate of the forward scattering for most particles and experimental setups.

Other parameters can be similarly derived (Section S2). For example, *R*_g_ can be estimated from the *I*_*n*_ values as 

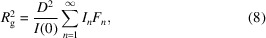

where 

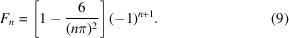




Another parameter describing particle size is the average vector length in the particle 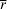 , which can be estimated from the *I*_*n*_ values as 

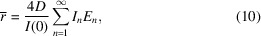

where 

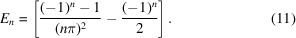




The Porod invariant *Q* is defined as the integrated area under the Kratky plot (Porod, 1982 ▸), which can be described in terms of the *I*_*n*_ values as 

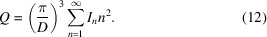

The Porod volume can then be calculated using the Porod invariant (Section S2) (Porod, 1982 ▸). The Porod volume is commonly used to estimate molecular weight for globular biological macromolecules. More recently, Rambo & Tainer (2013 ▸) derived a new SAS invariant termed the volume of correlation, *V*_c_, with units of length^2^ and which is related to the correlation length of the particle ℓ_c_. *V*_c_ can be used to estimate the molecular weight for macromolecules that may be either globular or flexible (Rambo & Tainer, 2013 ▸). *V*_c_ can be estimated from the *I*_*n*_ values as 

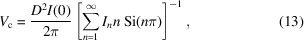

where Si(*n*π) is the Sine integral. The correlation length can similarly be calculated as 

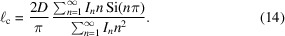

Since the variances and covariances of the *I*_*n*_ values are known from the least-squares minimization, error propagation can be used to determine the associated uncertainties for each of the parameters described above (Section S2).

### 2.3. Regularization of *P*(*r*)

The original IFT proposed by Glatter (1977 ▸) and other IFTs (Svergun, 1992 ▸; Vestergaard & Hansen, 2006 ▸) make use of regularization of the *P*(*r*) curve, similar to the general method of Tikhonov regularization (Tikhonov & Arsenin, 1977 ▸). The goal is to use the knowledge that *P*(*r*) functions are smooth for most particle shapes to generate curves that are free of strong oscillations from series termination and are relatively stable to statistical errors. Rather than minimize χ^2^ directly, a new function *T* is minimized, taking into account the smoothness of the *P*(*r*) curve according to equation (15): 

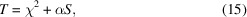

where *S* is the regularizing function, which can take different forms, and α is a Lagrange multiplier that acts as a weight to determine the strength of the smoothing. Larger α leads to a smoother *P*(*r*) function but may result in a worse fit of *I*(*q*) to the experimental data. The IFT method used by Moore has been shown to be more susceptible than other IFT methods to oscillations in the *P*(*r*) curve (Hansen & Pedersen, 1991 ▸; Svergun & Pedersen, 1994 ▸), most likely due to the lack of a regularizing function. We provide a detailed derivation of an analytical regularization of *P*(*r*) using *I*_*n*_ values in Section S3.

As for other similar IFT methods utilizing regularization, a suitable choice of α must be found to optimize the smoothness of the *P*(*r*) curve and the fit to the experimental data. Various methods for selecting the optimal value for α have been proposed, including via point of inflection (Glatter, 1977 ▸), Bayesian methods (Vestergaard & Hansen, 2006 ▸) and using perceptual criteria (Svergun, 1992 ▸). We describe our approach in Section 2.4 below.

Equation (3) assumes a sum from *n* = 1 to infinity. However, data are only collected to the maximum *q* value allowed by the experiment, *q*_max_. The lack of data for *q* > *q*_max_ implicitly corresponds to setting the *I*_*n*_ values to zero for those data points where *n* > *n*_max_ [where *n*_max_ = int(*q*_max_ *D*/π), *i.e.* the largest index in the series]. The regularization often results in poorer fits of the intensity profile at higher experimental *q* values with increasing α due to this implicit bias of *I*_*n*_ values for *n* > *n*_max_ towards zero. In order to remove this bias and allow for the *I*_*n*_ values at *n* > *n*_max_ to be unrestrained, *I*_*n*_ values for *n* > *n*_max_ are allowed to float (calculated up to 3*n*_max_). Note that the number of Shannon channels that can be reliably extracted from the data is dictated largely by the quality of the data in addition to the *q* range, as described by Konarev & Svergun (2015 ▸).

### 2.4. Implementation

**Figure 3:**
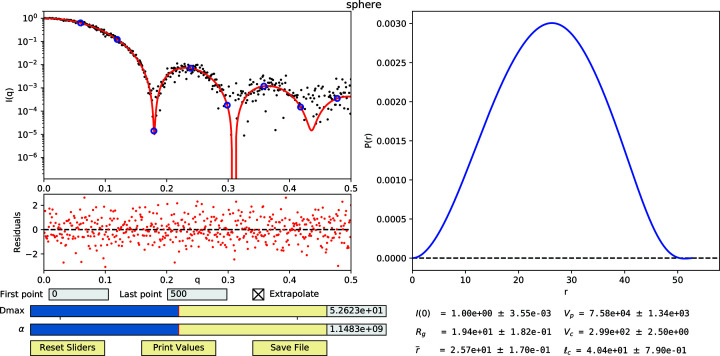
The interactive GUI display from the denss.f it_data.py script. The upper left panel shows the experimental *I*(*q*) curve as black circles, fitted *I*_*n*_ values as blue circles and the fitted *I*_c_(*q*) calculated from the *I*_*n*_ values as a red curve, all on a semilog plot. The residuals of the experimental and calculated intensity curves are shown below the intensity plot. The panel on the right shows the *P*(*r*) curve calculated from the *I*_*n*_ values. Input text boxes are provided at the bottom left to allow for trimming data points at the beginning or end of the curve, along with a checkbox to disable the calculation of intensities at high *q* values. Interactive sliders for *D*_max_ and α are also provided, along with corresponding input boxes for manual entry. The bottom right of the window shows size parameters calculated from the *I*_*n*_ values and associated uncertainties. Buttons for resetting the sliders, printing the size parameters and saving the results can be found at the bottom left.

Tools for performing the least-squares fitting of *I*_*n*_ values to experimental data, calculation of parameters and errors, and regularization of *P*(*r*) have been developed using Python, *NumPy* and *SciPy* (Harris *et al.*, 2020 ▸; Virtanen *et al.*, 2020 ▸) and are provided open source through the *DENSS* suite of SAS tools (Grant, 2018 ▸; https://github.com/tdgrant1/denss). The primary interface to use this algorithm is the denss.f it_data.py Python script. To enable ease of use, in addition to the command line interface, an interactive graphical user interface (GUI) (Fig. 3 ▸) has been developed using the *Matplotlib* package (Hunter, 2007 ▸).

#### 2.4.1. Automatic estimation of *D*

To assist users, upon initialization of the script the experimental data are loaded and estimates of *D* and α are automatically calculated. To estimate *D* automatically, an initial estimate of *D* is calculated that is likely to be significantly larger than the actual *D*. This subsequently enables a more accurate estimation of *D* where *P*(*r*) falls to zero. An initial value of *D* = 7*R*_g_ is used as this should ensure a large enough value given a variety of particle shapes (Petoukhov *et al.*, 2007 ▸; Grant *et al.*, 2015 ▸). An initial rough estimate of *R*_g_ is first calculated using the Guinier equation (Guinier *et al.*, 1955 ▸) with the first 20 data points. In cases where that estimate fails (*e.g.* due to excessive noise or a positive slope of the Guinier plot), the Guinier peak method is instead used (Putnam, 2016 ▸). The *I*_*n*_ values are then calculated from the experimental data using the regularized least-squares approach outlined in Section S3, setting α = 0 to optimize the fit to the data. After the initial *I*_*n*_ values have been calculated, the corresponding *P*(*r*) function often suffers from severe ripples caused by Fourier termination effects due to the finite range of data, as described above, making it difficult to estimate *D* where *P*(*r*) falls to zero. To alleviate this effect, a Hann filter, which is a type of Fourier filter (Blackman & Tukey, 1958 ▸), is applied to remove the Fourier truncation ripples from *P*(*r*). *D* is then calculated from this filtered *P*(*r*) curve as the first position *r* where *P*(*r*) falls below 0.01*P*_max_ after the maximum, where *P*_max_ is the maximum value of the filtered *P*(*r*). This new *D* value is then used to recalculate the *I*_*n*_ values for the best fit to the experimental scattering profile. In addition to automatically estimating *D* directly from the data, users can manually enter an initial estimate of *D* to begin with.

#### 2.4.2. Automatic estimation of α

Next, the optimal α is estimated, which yields *I*_*n*_ values corresponding to a smooth *P*(*r*) function while still resulting in a calculated *I*(*q*) curve that fits the experimental data. First, the best χ^2^ value possible is calculated by setting α = 0 and using the *D* value estimated in the previous step. Then, various values of α are scanned, from 10^−20^ to 10^20^ in logarithmic steps of 10^1^. This wide range is used to accommodate a variety of different scattering profiles covering a range of signal-to-noise values. At each step the χ^2^ is calculated. The optimal α is chosen by interpolating where 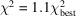 , *i.e.* where χ^2^ rises to 10% above the best possible value.

#### 2.4.3. Interface

The GUI mode of the script displays a plot of the intensities on a semilog *y* axis and plots the experimental data *I*_e_(*q*) and the initial fit *I*_c_(*q*), calculated from the *I*_*n*_ values at the experimental *q* (Fig. 3 ▸). The script additionally calculates *I*_c_(*q*) at *q* values extrapolated to *q* = 0. Users can alternatively provide a set of desired *q* values to calculate *I*_c_(*q*) as an ASCII text file when starting the program. The residuals, [*I*_e_(*q*_*i*_) − *I*_c_(*q*_*i*_)]/σ_*i*_, are also displayed to assist in assessing the quality of the fit. Next to the plot of intensities, the *P*(*r*) curve calculated from the *I*_*n*_ values is also displayed. In addition to input text boxes for manually entering new *D* and α values in the GUI, interactive sliders are available to change the *D* and α values, which automatically update the plots as they are adjusted. Users can also change the beginning and ending data points if desired, to remove outlier data points that often occur at either end of the experimental profile, or disable the calculation of intensities for *q* > *q*_max_. Several of the parameters described above, including *I*(0), *R*_g_, 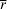 , *V*_p_, *V*_c_ and ℓ_c_, along with associated uncertainties, are calculated from the *I*_*n*_ values and displayed in the GUI. These parameters are updated interactively whenever *D* or α are changed.

#### 2.4.4. Calculation of *V*_p_, *V*_c_ and ℓ_c_

Particular care must be taken when estimating parameters that are sensitive to systematic errors in high-*q* data points, such as *V*_p_, *V*_c_ and ℓ_c_. In practice, direct estimation of these parameters using the equations described above may yield unstable results, even with regularization. Porod’s law is based on the assumption that all scattering comes from the surface of a particle, resulting in an asymptotic intensity decay proportional to *q* ^−4^ (Porod, 1982 ▸), giving rise to the ability to estimate values such as the Porod volume *V*_p_. In practice, shape scattering contributes significantly (Rambo & Tainer, 2011 ▸), as do systematic errors caused by inaccurate background subtraction (Manalastas-Cantos *et al.*, 2021 ▸), resulting in poor estimation of these parameters without correction. To deal with this, many algorithms impose an artificial constant subtraction to force the Porod decay, which has proven effective at providing accurate estimates of particle volume (Manalastas-Cantos *et al.*, 2021 ▸). However, different algorithms have different methods for calculating the constant to subtract and for determining the fitting region where these calculations are performed, and there is often subjectivity involved in selecting the appropriate ‘Porod region’ (Rambo & Tainer, 2011 ▸; de Oliveira Neto *et al.*, 2021 ▸). To avoid such issues with constant subtraction altogether, we have developed a different approach.

In our approach, we take advantage of the regularization provided above by intentionally oversmoothing using a large α. Oversmoothing has the effect of removing shape scattering while simultaneously enforcing a decay similar to Porod’s law of *q* ^−4^, making the resulting scattering profile more consistent with the assumptions of the Porod law. To do this, we multiply α by a factor of 10, which in our tests with experimental data resulted in the most accurate and robust results (see *Results* [Sec sec3] section below). We also limit the *q* range to 8/*R*_g_, which has previously been shown to be a reasonable cutoff for calculating Porod volume (Manalastas-Cantos *et al.*, 2021 ▸; de Oliveira Neto *et al.*, 2021 ▸). Note that this oversmoothing is only applied for calculation of the three parameters mentioned above and their associated errors and does not affect the actual fit of the scattering profile, *P*(*r*) curve or other parameters.

#### 2.4.5. Output

Finally, upon exiting the script, the experimental data and calculated fit of the intensities are saved in a file, with the calculated parameter values saved in the header. The corresponding *P*(*r*) curve is also saved.

In addition to providing the denss.f it_data.py script as an interface to the algorithm described above, other scripts in the *DENSS* package also utilize this algorithm, including denss.py and denss.all.py, to allow automatic fitting of the data and estimation of *D* and α when using these programs for *ab initio* 3D density reconstructions.

## 3. Results

One of the few shapes for which an analytical scattering equation has been derived is the solid sphere (Rayleigh, 1910 ▸; Porod, 1982 ▸). Since the equation of scattering for a sphere is known exactly, the *I*_*n*_ values for a sphere can be calculated directly (Section S4), resulting in equation (16), 

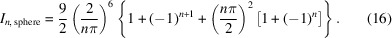

Note that the radius *R* of the sphere does not enter into equation (16). Interestingly, the odd *I*_*n*_ values for a sphere decay exactly as *q* ^−6^ and the even *I*_*n*_ values decay exactly as *q* ^−4^. The decay of intensity at higher angles proportional to *q* ^−4^ is described by Porod’s law as mentioned above, generally an approximation for most globular particles but here derived analytically for a sphere for even *I*_*n*_ values.

**Figure 4:**
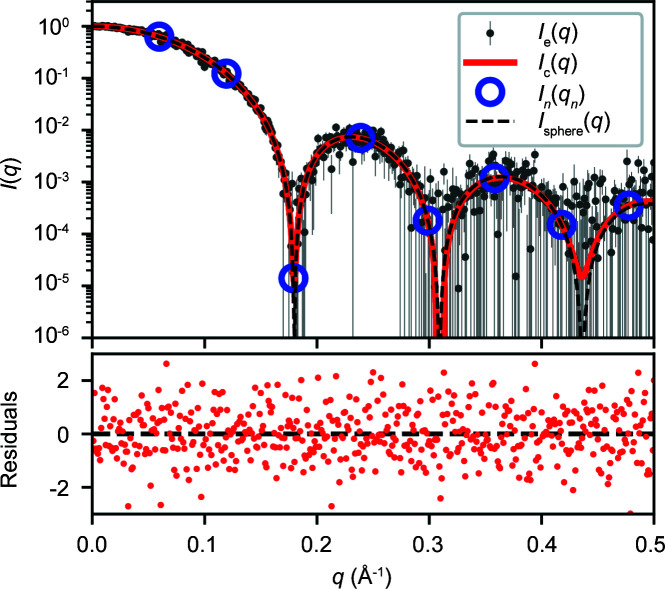
A plot of a calculated intensity curve *I*_c_(*q*) (red line) fitted to simulated noisy intensity values *I*_e_(*q*) (grey dots with error bars) for a sphere of radius 25 Å. The blue circles show the Shannon intensities *I*_*n*_(*q*_*n*_) and the black dashed line shows the exact scattering profile of the sphere *I*_sphere_(*q*). The bottom plot shows the residuals of the experimental data with respect to the calculated profile.

**Figure 5:**
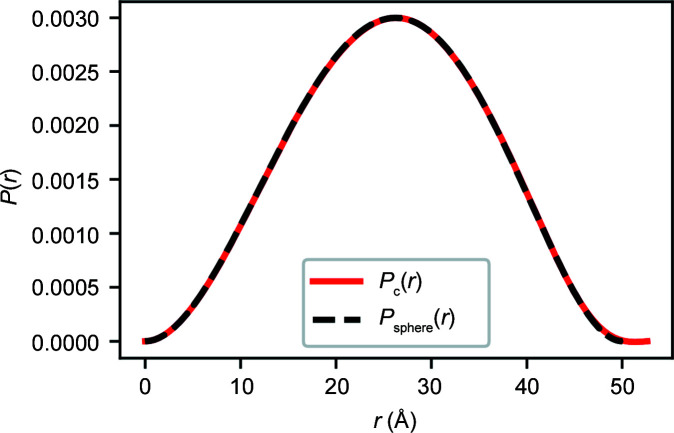
A plot of a calculated *P*_c_(*r*) curve from *I*_*n*_ values fitted to simulated noisy intensity values for a sphere of radius 25 Å. The exact *P*(*r*) curve for the sphere, *P*_sphere_(*r*), is also plotted as a dashed line.

All parameters outlined above, including *R*_g_, volume *etc.*, can be calculated analytically using equation (16), resulting in well known equations for solid spheres (Section S4). In Fig. 4 ▸ the scattering profile for a sphere of radius 25 Å with added Gaussian noise [*I*_e_(*q*)] is shown with the fitted *I*_*n*_ values and the recovered *I*_c_(*q*) profile. Eight Shannon points were used to fit the data, from which size parameters were calculated using the fitted *I*_*n*_ values, shown in Table 1 ▸. The *I*_*n*_ values can also be used to calculate the *P*(*r*) curve *P*_c_(*r*), shown in Fig. 5 ▸ along with the exact *P*(*r*) curve for a sphere (Porod, 1982 ▸) (Section S4).

**Figure 6:**
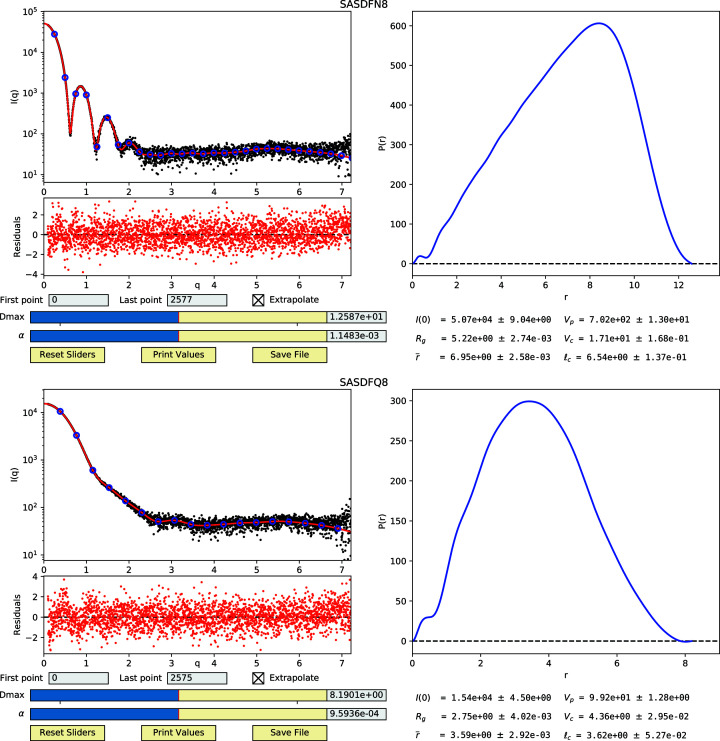
Fitting of *I*_*n*_ values to real experimental data sets while using regularization results in good quality fits, smooth *P*(*r*) curves and accurate parameter estimation. GUI displays are given for SASDFN8 (top) and SASDFQ8 (bottom). Note that the experimental *q* values were given in nanometres, resulting in nanometre units for parameters displayed.

Data from publicly accessible databases for experimental SAS data, such as BIOISIS (https://www.bioisis.net) and SASBDB (Valentini *et al.*, 2014 ▸), are particularly useful for verification and testing of algorithms such as that described here. To test denss.f it_data.py on experimental data sets, we downloaded two data sets from the benchmark section of the SASBDB online database, in particular SASDFN8 (apoferritin) and SASDFQ8 (bovine serum albumin) (Graewert *et al.*, 2020 ▸). Automated estimates of *D* and α were suitable for accurate fitting and parameter estimation, as indicated by the plot of residuals and comparison with the published parameter values (Fig. 6 ▸). Best fits are achieved when setting α = 0, as expected, and increasing α results in smoother *P*(*r*) plots. High-quality fits and smooth *P*(*r*) curves can be obtained simultaneously with an appropriate α (Fig. 6 ▸), while setting α to too large a value results in poorer fits to the intensity profile. Similar to other IFT methods, a balance must be struck to select the optimal α value resulting in the smoothest *P*(*r*) function possible while still enabling a good quality fit of *I*(*q*).

To compare the parameter estimates with other software, we used *DATGNOM* from the *ATSAS 3.0* package to estimate *R*_g_ and *I*(0), *DATPOROD* to estimate *V*_p_, and *DATVC* to estimate *V*_c_ from these two data sets (Manalastas-Cantos *et al.*, 2021 ▸). A comparison of parameter values calculated by *DATGNOM*/*DATPOROD*/*DATVC* and denss.f it_data.py is shown in Table 2 ▸. Overall, and very importantly for community standards, the values are similar for the two different methods [∼0.1% difference for *R*_g_ and *I*(0), and ∼3% difference for *V*_p_ and *V*_c_]. To verify that the error bounds are estimated correctly, we followed the protocol outlined by Manalastas-Cantos *et al.* (2021 ▸) to use the *DATRESAMPLE* program to generate 1000 resampled scattering profiles from the two SASBDB data sets. This allows the calculation of parameters from each resampled profile and subsequently an estimate of the statistical errors based on the standard deviation of the parameter values, for comparison with the errors estimated by the programs. The results of this analysis are also shown in Table 2 ▸. The analysis shows that denss.f it_data.py produces similar or smaller statistical errors compared with the estimated errors, suggesting the estimated errors should be considered an upper bound and the statistical errors probably less, whereas the statistical errors appear to be underestimated by *DATGNOM* [note that only *R*_g_ and *I*(0) have estimated errors reported]. It is noteworthy that the statistical errors on *R*_g_ and *I*(0) are smaller from denss.f it_data.py (two- to fivefold smaller) than from *DATGNOM*, while the statistical errors on *V*_p_ and *V*_c_ are about twofold smaller from *DATGNOM*/*DATPOROD*/*DATVC*.

**Figure 7:**
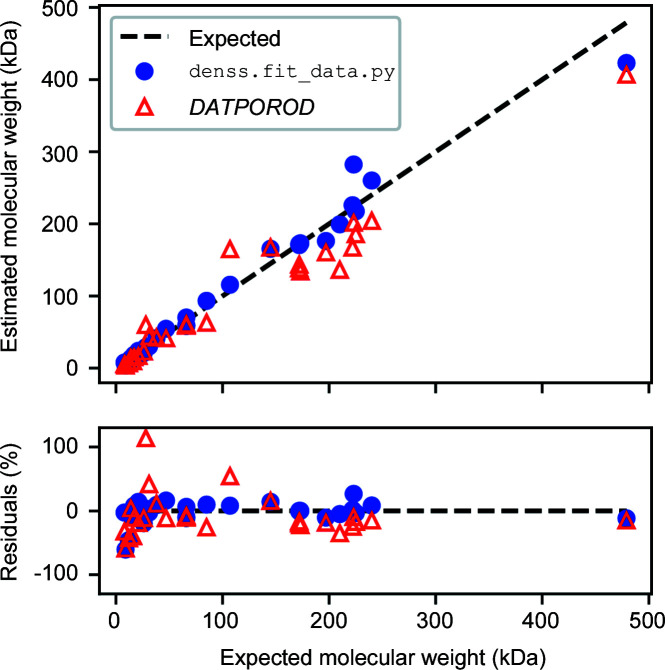
Comparison of expected molecular weight with values calculated using *V*_p_ estimated from denss.fit_data.py and *DATPOROD*.

The statistical errors described here are only based on resampling the scattering profile and do not account for systematic error that is likely to dominate. As discussed above, *V*_p_, *V*_c_ and ℓ_c_ are particularly sensitive to systematic deviation. To test the algorithm for accuracy with experimental data, we calculated *V*_p_ values for 29 data sets from the *Benchmark* section of the SASBDB and used *V*_p_ to estimate the molecular weight (MW) of the particle (where MW = *V*_p_/1.6). Fig. 7 ▸ shows a comparison of molecular weight values calculated using *V*_p_ estimates from denss.f it_data.py and *DATPOROD* with their expected values. Here, the expected value is taken from the expected molecular weight in the SASBDB entries calculated from the amino acid sequence. The median error from denss.f it_data.py is 8.7% and from *DATPOROD* is 18.0%. As expected, these real errors are in practice significantly larger than the <2% statistical or estimated errors in Table 2 ▸, confirming that systematic deviations dominate actual estimates of Porod volume from experimental data.

## 4. Discussion and conclusions

The approach outlined above is an extension of Moore’s original description of SAS profiles using a trigonometric series with the advantage of replacing the nondescript Moore coefficients with specific intensity values. As such, this derivation is subject to all of the same requirements as Moore’s, including the need for accurate intensity measurements for at least the first three Shannon channels to obtain reliable estimates of parameter values. We have described a derivation for performing regularization of the real-space *P*(*r*) curve analytically, and procedures for the automatic estimation of *D* and α values. We also present a novel approach for estimating parameters that are particularly sensitive to systematic deviations at high *q* values, such as *V*_p_.

As in Moore’s original approach, the use of least-squares minimization for the derivation given here of a series of SAS parameters directly from the *I*_*n*_ values has enabled the estimation of uncertainties through error propagation while accounting for covariances in the data. The oversampling of the information content in the SAS profile effectively increases the signal-to-noise ratio of each of the unique observations in the data, *i.e.* the *I*_*n*_ values. Additionally, the analytical regularization derived here simultaneously enables smooth *P*(*r*) curves and accurate fits to experimental data, all while providing error estimates for the *I*_*n*_ values and associated parameter calculations, accounting for covariances in the data. Using simulated and experimental data, we have shown that these methods yield parameter values describing the size and shape of particles that are as accurate as, and often more accurate than, existing tools.

The algorithm has been made available open source as a script called denss.f it_data.py, accessible on GitHub at https://github.com/tdgrant1/denss. The software can be run either from the command line or as an interactive GUI.

## 5. Related literature

The following additional references are cited in the supporting information: Fubini (1907 ▸); Tonelli (1909 ▸).

## Supplementary Material

Additional derivations. DOI: 10.1107/S1600576722006598/vg5144sup1.pdf

## Figures and Tables

**Table 1:** Parameters calculated from *I_n_* values for the sphere profile shown in Fig. 4 ▸ The columns correspond to expected parameter values using an infinite number of Shannon channels and the recovered values calculated from the fit.

Parameter	Expected	Calculated
*I*(0)	1.00	1.00 ± 0.004
*R*_g_ (Å)	19.36	19.38 ± 0.18
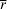 (Å)	25.71	25.74 ± 0.17
*V*_p_ (Å^3^)	65450	75838 ± 1338
*V*_c_ (Å^2^)	277.78	298.52 ± 2.50
ℓ_c_ (Å)	37.50	40.43 ± 0.79

**Table 2:** Comparison of parameter values calculated from experimental data sets SASDFN8 (DFN8) and SASDFQ8 (DFQ8) using *DATGNOM*/*DATPOROD*/*DATVC* or denss.f it_data.py Columns correspond to the value calculated for each parameter (Value) and either the estimated [± (Est.)] or statistical [± (Stat.)] errors as described in the text.

		DATGNOM/DATPOROD/DATVC			denss.f it_data.py		
Data set	Parameter	Value	± (Est.)	± (Stat.)	Value	± (Est.)	± (Stat.)
DFN8	*R*_g_ (nm)	5.216	5.451 × 10^−4^	2.931 × 10^−3^	5.222	2.835 × 10^−3^	5.933 × 10^−4^
DFN8	*I*(0)	5.063 × 10^4^	9.836	2.306 × 10^1^	5.070 × 10^4^	9.173	9.061
DFN8	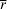 (nm)	N/A	N/A	N/A	6.950	2.644 × 10^−3^	7.212 × 10^−4^
DFN8	*V*_p_ (nm^3^)	6.704 × 10^2^	N/A	1.905	7.020 × 10^2^	1.303 × 10^1^	3.752
DFN8	*V*_c_ (nm^2^)	1.714 × 10^1^	N/A	8.717 × 10^−3^	1.709 × 10^1^	1.678 × 10^−1^	2.065 × 10^−2^
DFN8	ℓ_c_ (nm)	N/A	N/A	N/A	6.540	1.374 × 10^−1^	2.735 × 10^−2^
							
DFQ8	*R*_g_ (nm)	2.745	1.442 × 10^−3^	4.122 × 10^−3^	2.748	4.199 × 10^−3^	1.135 × 10^−3^
DFQ8	*I*(0)	1.542 × 10^4^	5.665	1.017 × 10^1^	1.542 × 10^4^	4.570	4.596
DFQ8	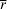 (nm)	N/A	N/A	N/A	3.594	3.010 × 10^−3^	1.164 × 10^−3^
DFQ8	*V*_p_ (nm^3^)	9.769 × 10^1^	N/A	3.367 × 10^−1^	9.917 × 10^1^	1.278	4.373 × 10^−1^
DFQ8	*V*_c_ (nm^2^)	4.638	N/A	2.890 × 10^−3^	4.355	2.949 × 10^−2^	4.579 × 10^−3^
DFQ8	ℓ_c_ (nm)	N/A	N/A	N/A	3.624	5.274 × 10^−2^	1.227 × 10^−2^
