# Supplementary material for: Describing small-angle scattering profiles by a limited set of intensities
Source: J Appl Crystallogr. 2022 Aug 30;55(Pt 5):1116–24. doi: 10.1107/S1600576722006598 (PMC9533761; doi:10.1107/S1600576722006598)
Supplement: Supplementary file 1 [file j-55-01116-sup1.pdf]

JOURNAL OF
APPLIED
CRYSTALLOGRAPHY

Volume 55 (2022)

Supporting information for article:

Describing small-angle scattering profiles by a limited set of intensities

Thomas D. Grant

Supporting Information

S1. Extension of Moore's IFT

Moore uses a trigonometric series to define a function $Q(r) = P(r)/r$. This definition resulted in a convenient relationship between the real space $Q(r)$ and the reciprocal space $U(q) = qI(q)$, where the two are Fourier mates. This results in equations 17 through 18 defining $P(r)$ and $I(q)$:

$$P(r) = \frac{r}{2\pi^2} \sum_{n=1}^{\infty} a_n \sin\left(\frac{n\pi r}{D}\right) \tag{17}$$

$$I(q) = \frac{D}{\pi} \sum_{n=1}^{\infty} \frac{a_n}{q} \left[\frac{\sin(qD - n\pi)}{qD - n\pi} - \frac{\sin(qD + n\pi)}{qD + n\pi}\right] \tag{18}$$

where a_n are weights for each term in the series, the Moore coefficients, and D is the maximum particle dimension (Note: modest variations compared to Moore's original description of these functions by a factor of 2π are due the use of $q = 4\pi \sin(\theta)/\lambda$ rather than $s = 2\sin(\theta)/\lambda$, where 2θ is the scattering angle and λ is the wavelength). Key to Moore's approach (and other IFT methods (Glatter, 1977; Svergun, 1992)) is that the weights a_n define both the real space and reciprocal space profiles, using the appropriate basis functions. Least squares can be used to determine the a_n's and the associated standard errors by minimizing the χ^2 formula (equation 19):

$$\chi^2 = \sum_{i=1}^{N} \left(\frac{I_e(q_i) - I_c(q_i)}{\sigma_i}\right)^2 \tag{19}$$

where I_e is the experimental intensity for data point i, I_c is the intensity calculated at q_i given by equation 18, σ_i is the experimental error on the intensities, and N is the total number of data points.

Moore's use of Shannon information theory to define $I(q)$ resulted in a selection of q values, namely $q_n = n\pi/D$, termed "Shannon channels" (Feigin & Svergun, 1987; Svergun & Koch, 2003; Rambo & Tainer, 2013). The intensities at q_n, i.e. $I_n = I(q_n)$, therefore become important values as they determine the a_n's and thus can be used to completely describe the low-resolution size and shape of a particle obtainable by SAS. It is therefore convenient to derive the mathematical relationship between I_n and a_n. Note that here we will further use m to refer to a particular term in the series, and we will use n when referring to the terms in the function defining the entire series. The intensity I_m at $q_m = m\pi/D$ is

$$I_m = I(q_m) = \frac{D}{\pi}\sum_{n=1}^{\infty}\frac{a_n}{m}\frac{D}{\pi}\left[\frac{\sin((n-m)\pi)}{(n-m)\pi} - \frac{\sin((n+m)\pi)}{(n+m)\pi}\right]. \tag{20}$$

Since

$$\left[\frac{\sin((n-m)\pi)}{(n-m)\pi} - \frac{\sin((n+m)\pi)}{(n+m)\pi}\right] = \begin{cases} 0 : n \neq m \\ 1 : n = m \end{cases} \tag{21}$$

the sum reduces to a single term when $m = n$, resulting in

$$I_m = \left(\frac{D}{\pi}\right)^2 \frac{a_m}{m} \tag{22}$$

and therefore

$$a_m = \left(\frac{\pi}{D}\right)^2 m I_m. \tag{23}$$

Equation 23 defines a relationship between the mth Moore coefficient and the intensity at the mth Shannon point. Inserting equation 23 into equation 18 and simplifying yields a general equation for $I(q)$ as a function of the intensity values at the Shannon points:

$$I(q) = 2\sum_{n=1}^{\infty} I_n \frac{(n\pi)^2}{(n\pi)^2 - (qD)^2}\frac{\sin(qD)}{qD}(-1)^{n+1}. \tag{24}$$

Defining the basis functions B_n as

$$B_n(q) = \frac{(n\pi)^2}{(n\pi)^2 - (qD)^2} \frac{\sin(qD)}{qD} (-1)^{n+1} \tag{25}$$

$I(q)$ can now be expressed as a sum of the basis functions B_n weighted by physical intensity values at q_n

$$I(q) = 2 \sum_{n=1}^{\infty} I_n B_n(q). \tag{26}$$

As in Moore's original approach, the B_n functions are determined by the maximum dimension of the particle, D. B_n's for $D = 50$ Å are illustrated in Figure 1. The $P(r)$ function can be determined from the continuous $I(q)$ according to equation 27:

$$P(r) = \frac{1}{2\pi^2} \int_{q=0}^{\infty} I(q) \frac{\sin(qr)}{qr} dq \tag{27}$$

$P(r)$ can also be represented using the series of I_n values by inserting equation 23 into equation 17, resulting in equation 28:

$$P(r) = \frac{r}{2D^2} \sum_{n=1}^{\infty} I_n n \sin\left(\frac{n\pi r}{D}\right) \tag{28}$$

or by defining real space basis functions S_n as follows:

$$P(r) = \sum_{n=1}^{\infty} I_n S_n(r) \tag{29}$$

$$S_n(r) = \frac{nr}{2D^2} \sin\left(\frac{n\pi r}{D}\right). \tag{30}$$

As intensity values measured precisely at each q_m are typically not collected during experiment, least squares minimization of χ^2 can be used to determine optimal values for each I_m from the oversampled SAS profile, resulting in greatly increased precision for each I_m compared to measured intensities. To determine the set of optimal I_m values, let

$$\chi^2 = \sum_{i=1}^{N} \frac{1}{\sigma_i^2} \left(I_e(q_i) - 2 \sum_{n=1}^{\infty} I_n B_n(q_i) \right)^2 \tag{31}$$

Values for each I_m are sought which minimize χ^2, i.e. where $\delta\chi^2/\delta I_m = 0$, yielding

$$\sum_i \frac{1}{\sigma_i^2} I_e(q_i) B_m(q_i) = 2 \sum_i \sum_n \frac{1}{\sigma_i^2} I_n B_m(q_i) B_n(q_i) \tag{32}$$

for all m. Let

$$Y_m = \sum_i \frac{1}{\sigma_i^2} I_e(q_i) B_m(q_i) \tag{33}$$

and

$$C_{mn} = 2 \sum_i \frac{1}{\sigma_i^2} B_m(q_i) B_n(q_i), \tag{34}$$

then $\boldsymbol{Y} = \boldsymbol{IC}$ and $\boldsymbol{I} = \boldsymbol{YC}^{-1}$, where $\boldsymbol{I}$ and $\boldsymbol{Y}$ denote the arrays of I_m and Y_m values, respectively, and $\boldsymbol{C}$ is a matrix whose elements are C_{mn}. The I_m's can now be used with equation 26 to provide a smoothed representation of $I(q)$ as well as $P(r)$ using equation 29. The matrix $\boldsymbol{C}^{-1}$ contains all the information on the variances and covariances of $\boldsymbol{I}$. The standard deviation for I_m can thus be calculated from $\boldsymbol{C}$ as

$$\sigma_m = \left[C_{mm}^{-1} \right]^{1/2}. \tag{35}$$

Furthermore the errors on the calculated $I_c(q)$ curve can be calculated as:

$$\sigma_{I_c(q)} = 2 \left[\sum_n \sum_m B_n(q) B_m(q) C_{nm}^{-1} \right]^{1/2} \tag{36}$$

and the errors in $P(r)$ are

$$\sigma_{P(r)} = \left[\sum_n \sum_m S_n(r) S_m(r) C_{nm}^{-1} \right]^{1/2}. \tag{37}$$

S2. Derivation of Size Parameters and Error Estimates

Here the detailed derivation is presented for calculating R_g from the I_n's. Derivations for the remaining parameters and errors can be determined similarly. R_g can be calculated from the $P(r)$ curve according to the following equation:

$$R_g^2 = \frac{\int r^2 P(r) dr}{2 \int P(r) dr}. \tag{38}$$

To determine the equation relating I_n coefficients to R_g, we substitute 28 into equation 38. Since

$$I(0) = 4\pi \int_0^D P(r)dr \tag{39}$$

the denominator can be simplified to

$$2\int_0^D P(r)dr = \frac{I(0)}{2\pi}. \tag{40}$$

The numerator becomes

$$\int_0^D r^2 P(r)dr = \int_0^D \frac{r^3}{2D^2} \sum_{n=1}^{\infty} I_n n \sin\left(\frac{n\pi r}{D}\right) dr. \tag{41}$$

Since Porod's law shows that intensity (and thus the I_n's) decays as q^{-4} for globular particles, and similarly as q^{-2} for random chains, the infinite sum is guaranteed to converge to a finite value. Since the sum converges, the Fubini and Tonelli theorems (Fubini, 1907; Tonelli, 1909) show that the infinite sum can be exchanged with the finite integral as follows

$$\int_0^D r^2 P(r)dr = \sum_{n=1}^{\infty} \int_0^D \frac{r^3}{2D^2} I_n n \sin\left(\frac{n\pi r}{D}\right) dr. \tag{42}$$

Pulling constants out of the integral results in

$$\int_0^D r^2 P(r)dr = \sum_{n=1}^{\infty} \frac{I_n n}{2D^2} \int_0^D r^3 \sin\left(\frac{n\pi r}{D}\right) dr. \tag{43}$$

The integral can be solved by three iterations of integration by parts and evaluated at the limits to obtain

$$\int_0^D r^3 \sin\left(\frac{n\pi r}{D}\right) dr = \frac{D^4}{n\pi}\left(1 - \frac{6}{(n\pi)^2}\right)(-1)^{n+1}. \tag{44}$$

Equation 44 can be combined with equation 43 and simplified to obtain the following equation for the numerator

$$\int_0^D r^2 P(r)dr = \sum_{n=1}^{\infty} \frac{D^2}{2\pi} I_n \left(1 - \frac{6}{(n\pi)^2}\right)(-1)^{n+1} \tag{45}$$

which can be combined with equation 40 to ultimately obtain equation 8. Similar steps can be followed for the remaining parameters.

The average vector length in the particle, $\overline{r}$, is defined as

$$\overline{r} = \frac{\int rP(r)dr}{\int P(r)dr}. \tag{46}$$

which can be combined with equation 28 to yield equation 10.

The Porod invariant, Q, is defined as the integrated area under the Kratky plot (Porod, 1982)

$$Q = \int_{q=0}^{\infty} q^2 I(q)dq \tag{47}$$

which, by plugging in equation 26, yields equation 12.

The Porod volume can then be calculated, using its definition containing the Porod invariant (Porod, 1982), by the following equation

$$V_p = \frac{2\pi^2 I(0)}{Q} \tag{48}$$

The Volume of Correlation (Rambo & Tainer, 2013), V_c, is defined as

$$V_c = \frac{I(0)}{\int qI(q)dq} = \frac{V_p}{2\pi\ell_c} \tag{49}$$

where ℓ_c is the length of correlation (Porod, 1982). V_c can thus be estimated from the I_n's according to equation 13. The length of correlation can be found from equation 49 or by combining equations 47, 48 and 49 it can be shown that

$$\ell_c = \pi \frac{\int qI(q)dq}{\int q^2 I(q)dq}. \tag{50}$$

Plugging in equation 26 into equation 50 yields equation 14.

Since the matrix $\boldsymbol{C}^{-1}$ contains all the information on the variances and covariances of the I_n's, the uncertainties in each parameter can be derived using error propagation. The error in $I(0)$ is thus

$$\sigma_{I(0)} = 2\left[\sum_n \sum_m (-1)^{n+m} C_{nm}^{-1}\right]^{1/2}. \tag{51}$$

The error in R_g is

$$\sigma_{R_g} = \frac{D^2}{2I(0)R_g}\left[\sum_n \sum_m F_n F_m C_{nm}^{-1}\right]^{1/2}. \tag{52}$$

In equation 8 it can be seen that R_g has a non-linear dependence on I_n, and thus the error on R_g is dependent on R_g itself. The error in $\overline{r}$ is

$$\sigma_{\overline{r}} = \frac{4D}{I(0)}\left[\sum_n \sum_m E_n E_m C_{nm}^{-1}\right]^{1/2}. \tag{53}$$

The error in Q is

$$\sigma_Q = \left(\frac{\pi}{D}\right)^3 \left[\sum_n \sum_m (nm)^2 C_{nm}^{-1}\right]^{1/2}. \tag{54}$$

The error in V_p is

$$\sigma_{V_p} = \frac{2\pi^2 I(0)}{Q^2}\sigma_Q. \tag{55}$$

The error in V_c is

$$\sigma_{V_c} = \frac{2\pi V_c^2}{D^2 I(0)}\left[\sum_n \sum_m nmSi(n\pi)Si(m\pi)C_{nm}^{-1}\right]^{1/2}. \tag{56}$$

The error in ℓ_c estimated from equation 49 becomes

$$\sigma_{\ell_c} = \frac{1}{2\pi V_c}\left[\sigma_{V_p}^2 + \left(\frac{V_p}{V_c}\right)^2 \sigma_{V_c}^2\right]^{1/2}. \tag{57}$$

S3. Derivation of Analytical Regularization of $P(r)$

To enable the regularization of the $P(r)$ curve for the derivation described above, S has been chosen to take the commonly used form of equation 58:

$$S = \int_0^D \left[P''(r)\right]^2 dr \tag{58}$$

where $P''(r)$ is the second derivative of $P(r)$ with respect to r. The second derivative is often chosen as it is sensitive to large oscillations in the $P(r)$ function, i.e. smoother functions will have fewer oscillations and thus S will be small. This representation

allows for an analytical solution to the problem of regularization of the $P(r)$ curve. To begin, the second derivative of $P(r)$ can be calculated as

$$P''(r) = \sum_{n=1}^{\infty} \frac{n^2\pi}{D^3} I_n \cos\left(\frac{n\pi r}{D}\right) - \sum_{n=1}^{\infty} \frac{n^3\pi^2}{2D^4} r I_n \sin\left(\frac{n\pi r}{D}\right). \tag{59}$$

Equation 58 requires squaring and then integrating equation 59, which is not possible analytically. However, ultimately we want to use least squares minimization of T in equation 15 to determine the optimal I_m values that yield the best fit of $I(q)$ to the experimental data and a smooth $P(r)$ function. The minimization of T with respect to I_m requires taking the derivative of S with respect to I_m. Therefore, rather than attempting to square and integrate equation 59, we can first take the derivative of S with respect to I_m as follows:

$$\begin{aligned}\frac{\delta S}{\delta I_m} &= \frac{\delta}{\delta I_m} \int_0^D \left[P''(r)\right]^2 dr \\ &= \int_0^D \frac{\delta \left[P''(r)\right]^2}{\delta I_m} dr \\ &= \int_0^D \frac{\delta}{\delta I_m} \left[\sum_{n=1}^{\infty} I_n \left(\frac{n^2\pi}{D^3} \cos\left(\frac{n\pi r}{D}\right) - \frac{n^3\pi^2}{2D^4} r \sin\left(\frac{n\pi r}{D}\right)\right)\right]^2 dr. \end{aligned} \tag{60}$$

Now we can take the derivative as

$$\frac{\delta S}{\delta I_m} = \int_0^D 2\left[\frac{m^2\pi}{D^3} \cos\left(\frac{m\pi r}{D}\right) - \frac{m^3\pi^2}{2D^4} r \sin\left(\frac{m\pi r}{D}\right)\right]\left[\sum_{n=1}^{\infty} I_n \left(\frac{n^2\pi}{D^3} \cos\left(\frac{n\pi r}{D}\right) - \frac{n^3\pi^2}{2D^4} r \sin\left(\frac{n\pi r}{D}\right)\right)\right] dr. \tag{61}$$

Since the term in square brackets outside the sum is independent of n, it can be brought inside the sum, and the integration and summation exchanged:

$$\begin{aligned}\frac{\delta S}{\delta I_m} &= \int_0^D 2\sum_{n=1}^{\infty} I_n \left[\frac{m^2\pi}{D^3} \cos\left(\frac{m\pi r}{D}\right) - \frac{m^3\pi^2}{2D^4} r \sin\left(\frac{m\pi r}{D}\right)\right]\left[\frac{n^2\pi}{D^3} \cos\left(\frac{n\pi r}{D}\right) - \frac{n^3\pi^2}{2D^4} r \sin\left(\frac{n\pi r}{D}\right)\right] dr \\ &= \sum_{n=1}^{\infty} I_n \int_0^D 2\left[\frac{m^2\pi}{D^3} \cos\left(\frac{m\pi r}{D}\right) - \frac{m^3\pi^2}{2D^4} r \sin\left(\frac{m\pi r}{D}\right)\right]\left[\frac{n^2\pi}{D^3} \cos\left(\frac{n\pi r}{D}\right) - \frac{n^3\pi^2}{2D^4} r \sin\left(\frac{n\pi r}{D}\right)\right] dr. \end{aligned} \tag{62}$$

The terms in the integrand can now be expanded, yielding four terms in total:

$$\begin{aligned}\frac{\delta S}{\delta I_m} = \sum_{n=1}^{\infty} I_n \int_0^D 2\Bigg[& \frac{m^2\pi}{D^3}\cos\left(\frac{m\pi r}{D}\right)\frac{n^2\pi}{D^3}\cos\left(\frac{n\pi r}{D}\right) \\ & - \frac{m^2\pi}{D^3}\cos\left(\frac{m\pi r}{D}\right)\frac{n^3\pi^2}{2D^4} r\sin\left(\frac{n\pi r}{D}\right) \\ & - \frac{m^3\pi^2}{2D^4} r\sin\left(\frac{m\pi r}{D}\right)\frac{n^2\pi}{D^3}\cos\left(\frac{n\pi r}{D}\right) \\ & + \frac{m^3\pi^2}{2D^4} r\sin\left(\frac{m\pi r}{D}\right)\frac{n^3\pi^2}{2D^4} r\sin\left(\frac{n\pi r}{D}\right)\Bigg] dr. \end{aligned} \tag{63}$$

Each term can now be integrated and evaluated at the limits to obtain the following:

$$\begin{aligned}\frac{\delta S}{\delta I_m} = \sum_{n=1}^{\infty} I_n \Bigg[& 0 \\ & - \frac{(-1)^{m+n} m^2 n^4 \pi^2}{2D^5(m^2 - n^2)} \\ & + \frac{(-1)^{m+n} m^4 n^2 \pi^2}{2D^5(m^2 - n^2)} \\ & - \frac{(-1)^{m+n} (mn)^2 (m^4 + n^4)\pi^2}{2D^5(m^2 - n^2)^2}\Bigg]. \end{aligned} \tag{64}$$

These terms can be combined and represented by G_{mn} below. Note that when $m = n$ the equation is undefined, so the integration has been repeated after taking the limits as m approaches n for the special case when $m = n$. Taken together, the derivative of S with respect to I_m can be now be represented as equation 65:

$$\frac{\delta S}{\delta I_m} = \sum_{n=1}^{\infty} I_n G_{mn} \tag{65}$$

where

$$G_{mn} = \begin{cases} \frac{\pi^2}{2D^5}(mn)^2 \frac{m^4 + n^4}{(m^2 - n^2)^2}(-1)^{m+n} & : m \neq n \\ \frac{\pi^2}{48D^5} n^4 \left(2n^2\pi^2 + 33\right) & : m = n \end{cases}.$$

Following the same procedure outlined in equations 31 through 34 and now including 65, equation 15 can now be solved by least squares minimization to yield the optimal values for each I_m while accounting for the regularizing function S according to the following modified equations:

$$\sum_i \frac{1}{\sigma_i^2} I_e(q_i) B_m(q_i) = \sum_{n=1}^{\infty} I_n \left[\alpha G_{mn} + 2\sum_i \frac{1}{\sigma_i^2} B_m(q_i) B_n(q_i)\right]. \tag{66}$$

Once again, letting

$$Y_m = \sum_i \frac{1}{\sigma_i^2} I_e(q_i) B_m(q_i) \tag{67}$$

and now letting

$$C_{mn} = \alpha G_{mn} + 2 \sum_i \frac{1}{\sigma_i^2} B_m(q_i) B_n(q_i) \tag{68}$$

then the matrix of I_m values, $\boldsymbol{I}$, can be found in a similar fashion as before:

$$\boldsymbol{I} = \boldsymbol{Y}\boldsymbol{C}^{\boldsymbol{-1}}. \tag{69}$$

S4. Derivation of Sphere Parameters

The equation governing the scattering of a sphere of radius R is given by equation 70 (Rayleigh, 1910; Porod, 1982):

$$I_{sphere}(q) = I(0) \left[3 \frac{\sin(qR) - qR\cos(qR)}{(qR)^3} \right]^2 \tag{70}$$

(for simplicity, $I(0)$ is here used as a global scale factor accounting for proportionality constants related to volume, concentration, scattering length density, etc., and is set to unity in the following equations). By evaluating equation 70 at $q_n = n\pi/D = n\pi/2R$, we obtain a representation of the intensity from a sphere as a function of I_n's, shown by equation 16. All of the parameters derived above can be expressed for a sphere as well, yielding well-known values for spheres of radius $R = D/2$. For example, equation 8 can be used to calculate the radius of gyration of a sphere of radius R by inserting equation 16 yielding

$$R_{g,sphere}^2 = D^2 \sum_{n=1}^{\infty} \frac{9}{2} \left(\frac{2}{n\pi} \right)^6 \left[1 + (-1)^{n+1} + \left(\frac{n\pi}{2} \right)^2 (1 + (-1)^n) \right] F(n) \tag{71}$$

The entire infinite sum converges to $\frac{3}{20}$ and the expression simplifies to

$$R_{g,sphere}^2 = \frac{3D^2}{20} = \frac{3}{5} R^2 \tag{72}$$

which is the well-known equation relating the radius of gyration of a solid sphere to its radius. Similarly, the Porod invariant Q of a sphere can be calculated according to equation 12 and shown to be

$$Q_{sphere} = \frac{12\pi}{D^3} = \frac{3\pi}{2R^3} \tag{73}$$

which can be combined with equation 48 to evaluate the Porod volume as

$$V_{p,sphere} = \frac{4\pi}{3} R^3 \tag{74}$$

which is the well-known equation for the volume of a sphere of radius R. Each of the other parameters listed in the equations above can also be solved in a similar fashion and shown to be as follows:

$$\overline{r}_{sphere} = \frac{36}{35} R \tag{75}$$

$$V_{c,sphere} = \frac{4}{9} R^2 \tag{76}$$

$$\ell_{c,sphere} = \frac{3}{2} R. \tag{77}$$

The $P(r)$ curve for a sphere has previously been derived (Porod, 1982):

$$P_{sphere}(r) = r^2 \left(1 - \frac{3}{2} \left(\frac{r}{D} \right) + \left(\frac{1}{2} \frac{r}{D} \right)^3 \right). \tag{78}$$
